# Dentists and dental hygienists’ comprehension of HIV infection associated periodontal implications and management

**DOI:** 10.3389/fpubh.2024.1370112

**Published:** 2024-04-04

**Authors:** Muzammil Moin Ahmed

**Affiliations:** Department of Public Health, College of Applied Medical Sciences, Qassim University, Buraydah, Al-Qassim, Saudi Arabia

**Keywords:** HIV, AIDS, infection prevention, periodontal diseases, infectious disease, periodontal implications, periodontal systemic diseases

## Abstract

**Background:**

In an era wherein, persuasive evidence continues to witness the association between systemic and periodontal diseases, the absence of scientific data on dental professionals’ comprehension concerning the HIV infection and periodontal link is lamentably backward. Thus, the key objective of this research is to ascertain the extent of comprehension possessed by dentists and dental hygienists concerning periodontal implications and their management in HIV patients.

**Methods:**

It is a quantitative cross-sectional survey employing a descriptive approach focusing on a specific cohort of dental professionals. The study setting featured an online platform for the distribution of concealed, closed-ended, structured questionnaire. The data was gathered for four sections: six comprehension statements about periodontal manifestations in HIV patients; fifteen comprehension statements about HIV patients’ periodontal management; eight familiarity statements about HIV management; and two educational statements about HIV. The comparisons of comprehension scores were drawn between variables such as specialties, age groups, and genders.

**Results:**

The survey represented 468 dental professionals representing distinct dental specialties, with a mean age of 24.26 ± 7.53 years. The mean comprehension score for all groups of participants is 10.31 ± 9.34 (33.25%). The highest scores were recorded among those aged 31–40 (20.67 ± 8.31), followed by those aged 40+ (19.38 ± 9.39), 20–30 (9.53 ± 8.96), and under 20 (8.92 ± 8.57), at *p* < 0.001. The female participants (15.06 ± 12.2) exhibited substantially better scores in contrast to the male participants (8.74 ± 7.57). Periodontists (27.77 ± 3.08) comprehended most, then the oral medicine practitioners (25 ± 0). Dental hygiene students (5.52 ± 3.56) and hygienists (7.67 ± 9.72) comprehended the least. The scores for all four domains assessed were disappointingly low: knowledge about HIV-periodontal manifestations (2.81 ± 2.18), knowledge about management of periodontal diseases in HIV patients (3.73 ± 4.7), familiarity with periodontal care in HIV patients (2.87 ± 3.01), and education received about HIV and periodontal diseases (0.91 ± 0.66).

**Conclusion:**

Dental professionals are notably incomprehensive, unfamiliar, and lacking in expertise in the realm of periodontal facets of HIV. The periodontists and oral medicine practitioners showed a substantial amount of comprehension, while the dental hygiene students and dental hygienists presented a conspicuously inadequate level of comprehension. The study outcome could potentially serve as an invaluable instrument for self-assessment by dental professionals and educators. HIV/AIDS ought not to persist as an unspoken taboo or disregarded subject within the dental field, particularly in periodontics, but rather should receive prominence in dental schools and professional development programs.

## Introduction

Human immunodeficiency virus (HIV) infection is one of the fatal conditions that affect the host immune system, depleting its immune cells. HIV infection over a protracted period of time results in the onset of a disease complex, the acquired immunodeficiency syndrome (AIDS). In the year 2021, there are 38.4 million individuals living with HIV, and to date, it has clutched the lives of 40.1 million individuals around the world; the number of fatalities caused by HIV is only anticipated to rise in the years to come (1). Numerous HIV-positive people are oblivious to the fact that they have the virus, which accounts for 40 % of all HIV exposures in the USA (2).

Depending on the CD4+ cell depletion rate, compromised host immunity caused by HIV infections can appear in a variety of ways, ranging from opportunistic bacterial and fungal infections to severe malignancies (3). Oral lesions caused by HIV can vary greatly in severity and frequency, but some are almost always present. One of the persistent findings in HIV is periodontal disease, observed even in individuals on antiviral treatment (4). HIV has the potential to either change the course of periodontal diseases that are already present or to trigger the development of more severe types of periodontal diseases. Some of the most prevalent presentations of HIV in the periodontium are HIV-associated periodontitis and gingivitis, linear gingival erythema, and necrotizing ulcerative gingivitis and periodontitis. The management of these periodontal lesions induced by HIV necessitates comprehensive periodontal debridement, an antimicrobial regimen, and effective supportive periodontal therapy (5). Not only does HIV influence the onset and course of periodontal diseases, but periodontal biofilm and its products also affect the advancement and treatment outcomes of HIV infection (6). Valentine et al. in 2016 observed an improvement in pro-inflammatory markers after the elimination of periodontal inflammation in HIV patients (7).

Although sexual contact and needle sharing with infected persons account for major modes of HIV transmission, the possibility of occupational exposure to healthcare providers cannot be dismissed (8, 9). In dental settings, the most prevalent ways for HIV to be spread are via needlestick injuries as well as through contact with contaminated fluids and splashes.

The papers that were published in the 1980s by Silverman et al. during the initial period of HIV discovery provide evidence that dental practitioners play a significant role in the early diagnosis of HIV infection (10). Additionally, dental practitioners play an integral part in the treatment of HIV-associated oral diseases and, as a result, in the improvement of the overall quality of life for HIV patients. The responsibility of implementing strategic methods for the management of HIV induced periodontal diseases and conditions lies with dental professionals, more specifically dental hygienists and periodontists.

In light of the fact that dentists and dental hygienists are crucial for the diagnosis, treatment, and prevention of HIV associated oral lesions, it’s essential for them to be conversant with the dental and periodontal implications of HIV, as well as the procedures and protocols that may be used while dealing with the cases of HIV that are recognized as well as those that are unknown. There is a substantial body of research on hand that examines dentists’ levels of knowledge and attitudes about HIV associated dental diseases and their management (11–14). Nevertheless, there is a paucity of investigations that assess comprehension of the HIV-related periodontal implications, particularly among dental hygienists. As a result, the primary purpose of this research is to assess the level of comprehension held by dentists and dental hygienists on the periodontal implications and their management in HIV.

## Materials and methods

### Study design

The research is characterized as a quantitative cross-sectional survey employing a descriptive approach focusing on a cohort of dental professionals. The research adhered to the World Medical Association’s latest amended Declaration of Helsinki ethical norms (15) and was granted approval by the institutional review board (No. 23-36-07).

### Study population

The participants included senior-level dentistry and dental hygiene students, currently enrolled in 12 dental and 4 dental hygiene institutes in different regions of Saudi Arabia. These institutes were selected based on their geographical representation of different regions in Saudi Arabia. Additionally, the study also involved general and specialty dental practitioners as well as dental hygiene practitioners working in 26 private and 59 government hospitals and polyclinics. The study excluded individuals who did not provide voluntary consent to participate and individuals who were below the age of 18.

### Sample size

The research was determined to contain 400 samples from four distinct groups: dentistry students, dental hygiene students, dental and specialist practitioners, and dental hygiene practitioners. To arrive at a confidence interval of 95%, the sample size was calculated using Cochran’s formula, in accordance with Bartlett et al. (16). The sampling strategy employed to recruit and distribute participants into dental professional groups was non-probability voluntary response sampling (17).

### Data collection methods

The study setting featured an online platform for the distribution of concealed, closed-ended, structured questionnaire. The dental and dental hygiene professionals were invited to participate in the survey through emails and WhatsApp. The questionnaire’s content validity was established through a process of peer review, wherein two periodontists evaluated the questions, and the construct validity was assessed by administering the questionnaire to a small sample (*N* = 50; 12.5%) prior to its administration to a larger sample to identify any necessary modifications, as reported by Tsang et al. (18). The 50 participants included for validity assessment comprised dental students (*n* = 10), dental hygiene students (*n* = 10), dental hygienists (*n* = 10), general dentists (*n* = 10), and specialists’ dentists (*n* = 10). Prior to conducting the validity assessment, the questionnaire contained response options of “strongly agree,” “agree,” “not sure,” “disagree,” and “strongly disagree.” The validity testing revealed that these response options caused confusion among the participants. Therefore, they were simplified by providing the choices of agree, neutral, and disagree.

### Study parameters and variables

The data was gathered for four sections of the questionnaire: the first section consisted of six comprehension statements pertaining to periodontal manifestations in patients with HIV; the second section included fifteen comprehension statements regarding the periodontal management of HIV patients; the third section comprised eight familiarity statements concerning the management of patients with HIV; and the fourth section focused on two educational statements pertaining to HIV and periodontal diseases. Participants were given the option of agreeing, disagreeing, or remaining neutral on each survey item. One point was considered for the correct response, while no points were considered for any that were incorrect. Age, gender, and dental professional subgroups of participants served as independent variables, while knowledge statements regarding periodontal manifestation and periodontal management of HIV patients, familiarity statements regarding HIV patient management, and education statements regarding HIV and periodontal disease served as dependent variables.

### Data analysis

The study employed descriptive and inferential statistics, delivering continuous measurements as mean ± SD (min-max) and categorical measurements as number (%) using SPSS 22.0 and R environment ver. 3.2.2 Comprehension scores were compared among the research participants. A one-way analysis of variance (ANOVA) was employed to assess the difference among the independent groups. The chi-square test was applied to ascertain the statistical significance of parameters measured on a categorical scale across different groups, whereas the Fisher exact test was utilized for small cell samples. All statistical tests were carried out following standards specified by Rosner et al. (19) and Riffenburg et al. (20).

## Results

**Figure 1:**
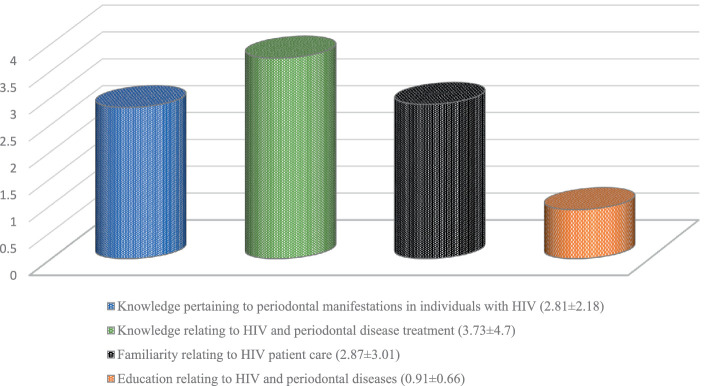
Mean comprehension scores for the four sections of questionnaire.

A total of 486 dental professionals participated in the study, surpassing the initially projected sample size, with a mean age of 24.26 ± 7.53 years. The information regarding the distribution of the subjects based on gender as well as the specialist group is shown in Table 1. The performance results of participants comprehension, familiarity, and education about human immunodeficiency virus-associated periodontal implications and management can be categorized into four levels: high (100–75%), average (74–50%), low (49–25%), and very low (24–0%) as presented in Table 2. The mean comprehension score for all groups of participants is 10.31 ± 9.34 (33.25%). Upon comparing the mean comprehensive scores among the various age groups of study participants, it emerged that those aged 31 to 40 had the highest scores (20.67 ± 8.31), followed by those aged 40 and older (19.38 ± 9.39), those aged 20 to 30 (9.53 ± 8.96), and those aged under 20 (8.92 ± 8.57), at *p* < 0.001. The female participants (15.06 ± 12.2) exhibited substantially better scores in comprehension in contrast to the male participants (8.74 ± 7.57). The questionnaire used for the research was made up of four distinct sections: 1. knowledge pertaining to periodontal manifestations in individuals with HIV; 2. knowledge relating to HIV and periodontal disease treatment; 3. familiarity relating to HIV patient care; and 4. education relating to HIV and periodontal diseases. Figure 1 depicts the findings of these four sections. Comparisons between different groups, such as dental students, dental hygiene students, dental hygienists, general dentists, dental specialists, and periodontists, are shown in Tables 3–6. Dental students (11.13 ± 10.44) have lower comprehension scores compared to general dentists (15.56 ± 8.12), and a similar pattern is seen between dental hygiene students (5.52 ± 3.56) and dental hygienists (7.67 ± 9.72). General dentists (11.11 ± 3.99) do not vary much from other dental specialists (12.25 ± 11.18) at *p* = 0.42. However, the periodontist (27.77 ± 3.08) demonstrated greater comprehension compared to other dental specialists (9.77 ± 8.25).

**Table 1:** Distribution of research subjects based on gender and specialty.

Specialty	Gender		Total	*p*-value
	Female	Male		
Dental students	34 (29.3%)	107 (30.4%)	141 (30.1%)	0.920
Dental hygienists	17 (14.7%)	87 (24.7%)	104 (22.2%)	0.033*
Dental hygiene students	40 (34.5%)	61 (17.3%)	101 (21.6%)	<0.001**
General dentists	5 (4.3%)	59 (16.8%)	64 (13.7%)	<0.001**
Periodontists	6 (5.2%)	20 (5.7%)	26 (5.6%)	1.000
Oral and maxillofacial surgeons	5 (4.3%)	8 (2.3%)	13 (2.8%)	0.324
Oral pathologists	4 (3.4%)	1 (0.3%)	5 (1.1%)	0.014*
Orthodontists	2 (1.7%)	3 (0.9%)	5 (1.1%)	0.601
Operative dentists	2 (1.7%)	2 (0.6%)	4 (0.9%)	0.257
Prosthodontists	0 (0%)	2 (0.6%)	2 (0.4%)	1.000
Pedodontists	1 (0.9%)	1 (0.3%)	2 (0.4%)	0.434
Oral medicine practitioners	0 (0%)	1 (0.3%)	1 (0.2%)	1.000
Total	116 (100%)	352 (100%)	468 (100%)	-

**Table 2:** Performance results of comprehension, familiarity, and education statements.

Comprehension, familiarity, and education statements	Subjects (*n*)	Comprehension ratio (%)	Performance interpretation
I learned about periodontal manifestations in HIV patients during my dental/dental hygiene study.	339	72.4	High
HIV infection can worsen existing periodontal diseases.	330	70.5	High
HIV infection can show characteristic periodontal features.	266	56.8	Average
I am familiar with possible HIV transmission modes during periodontal treatment.	238	50.9	Average
I am familiar with the scaling and root planning protocol for HIV patients.	236	50.4	Average
I am familiar with the necrotizing periodontal diseases management procedures in HIV patients.	235	50.2	Average
Necrotizing gingivitis and periodontitis are often seen in HIV.	229	48.9	Low
HIV typically appears as linear gingival erythema.	225	48.1	Low
HIV infection can cause new periodontal lesions.	182	38.9	Low
I am familiar with periodontal follow-up/maintenance mechanisms for HIV patients.	168	35.9	Low
I am familiar with the adjunctive periodontal antimicrobial regimen for HIV patients.	165	35.3	Low
Dental radiographs are safe in HIV patients.	163	34.8	Low
Linear gingival erythema should be treated as soon as it is detected.	161	34.4	Low
I am familiar with the post-exposure protocol after HIV exposure.	151	32.3	Low
Periodontal diseases in HIV are treated with conventional periodontal procedures.	148	31.6	Low
Antimicrobial mouthwashes are useful in HIV patients.	120	25.6	Low
Local anesthesia is safe in HIV patients.	89	19	Very low
Linear gingival erythema responds well to the standard periodontal therapy protocols.	86	18.4	Very low
I learned about periodontal management in HIV patients during my dental/dental hygiene study.	85	18.2	Very low
Periodontal diseases can affect HIV treatment outcomes.	84	17.9	Very low
Ultrasonic scaling is safe in HIV patients.	84	17.9	Very low
Linear gingival erythema responds well to the standard plaque control measures	83	17.7	Very low
Periodontal diseases severity has correlation with CD4+ cells depletion rate in HIV.	82	17.5	Very low
Root surface debridement is safe in HIV patients.	81	17.3	Very low
I am familiar with rapid HIV testing process in dental settings.	80	17.1	Very low
Special infection control measures should be adopted for HIV patients.	79	16.9	Very low
Full mouth periodontal probing is safe in HIV patients.	79	16.9	Very low
Prophylactic antibiotics should be avoided in HIV patients.	74	15.8	Very low
Periodontal treatment can reduce the progress of HIV infection.	70	15	Very low
I provided periodontal care to an HIV patient.	69	14.7	Very low
Periodontal treatment can improve CD4+ cells count in HIV.	65	13.9	Very low

**Table 3:** Comparison between dental students and general dentists and dental specialists together.

Section	Students/Dentists		*p*-value
	Dental students	General dentists and dental specialists	
1	2.79 ± 2.33	4.01 ± 1.63	<0.001
2	3.84 ± 5.32	5.8 ± 4.85	0.002
3	3.62 ± 2.9	4.51 ± 2.76	0.012
4	0.88 ± 0.71	1.24 ± 0.55	<0.001
Total	11.13 ± 10.44	15.56 ± 8.12	<0.001

1 – knowledge pertaining to periodontal manifestations in individuals with HIV; 2 – knowledge relating to HIV and periodontal disease treatment; 3 – familiarity relating to HIV patient care; 4 – education relating to HIV and periodontal diseases.

**Table 4:** Comparison between dental hygiene students with dental hygienists.

Section	Students/Dental hygienists		*p*-value
	Dental hygienists	Dental hygiene students	
1	1.91 ± 2.26	2.3 ± 1.8	0.180
2	2.57 ± 4.6	2.25 ± 2.06	0.524
3	2.57 ± 2.89	0.15 ± 0.99	<0.001
4	0.63 ± 0.73	0.83 ± 0.45	0.015
Total	7.67 ± 9.72	5.52 ± 3.56	0.038

1 – knowledge pertaining to periodontal manifestations in individuals with HIV; 2 – knowledge relating to HIV and periodontal disease treatment; 3 – familiarity relating to HIV patient care; 4 – education relating to HIV and periodontal diseases.

**Table 5:** Comparison between general dentists and dental specialists.

Section	General dentists/Dental specialists		*p*-value
	General dentists	All Specialists	
1	3.19 ± 1.6	2.99 ± 2.4	0.540
2	2.44 ± 2.12	5.06 ± 5.58	<0.001
3	4.48 ± 1.48	3.27 ± 3.33	0.006
4	1 ± 0.31	0.94 ± 0.81	0.554
Total	11.11 ± 3.99	12.25 ± 11.18	0.426

1 – knowledge pertaining to periodontal manifestations in individuals with HIV; 2 – knowledge relating to HIV and periodontal disease treatment; 3 – familiarity relating to HIV patient care; 4 – education relating to HIV and periodontal diseases.

**Table 6:** Comparison between periodontists and other dental professionals.

Section	Periodontists/Other dental professionals		*p*-value
	Periodontists	Other dental professionals	
1	5.42 ± 0.58	2.71 ± 2.14	<0.001
2	12.54 ± 2.86	3.19 ± 4.13	<0.001
3	7.85 ± 0.46	3.06 ± 2.72	<0.001
4	1.96 ± 0.2	0.82 ± 0.64	<0.001
Total	27.77 ± 3.08	9.77 ± 8.25	<0.001

1 – knowledge pertaining to periodontal manifestations in individuals with HIV; 2 – knowledge relating to HIV and periodontal disease treatment; 3 – familiarity relating to HIV patient care; 4 – education relating to HIV and periodontal diseases.

## Discussion

The subject focused on in this research is the comprehension of dental professionals regarding the periodontal consequences and care linked to HIV. The reason for selecting this topic, in addition to the lack of available data, is the crucial nature of acquiring necessary knowledge among dental professionals and the chances of attending HIV-positive patients, either in subclinical or symptomatic stages. Failure to comprehend the implications of such experiences may result in the failure to recognize these patients in dental offices, thereby posing a potential risk.

A relevant finding of this study was a notable lack of understanding (33.25%; a mean score of 10.31 ± 9.34) among dentists and dental hygienists on several facets of periodontal implications and care related to HIV. A multitude of studies have evaluated dental hygienists and dentists general understanding of HIV in highly regarded journals (11–14). On the other hand, there is not a single study that assesses understanding about the different periodontal aspects of HIV. This makes current research the first to explore these aspects.

Upon comparing the understanding ratings across different age groups, it was apparent that the young respondents exhibited the lowest level of comprehension. This finding aligns with the data presented in the worldwide surveillance report of UNAIDS, which indicates a deficiency in the understanding of HIV/AIDS among young individuals (21). In the present study, it was observed that female participants exhibited superior comprehension compared to their male counterparts. In this aspect, the literature is somewhat contentious, with some research supporting our finding and others contradicting it. The observation made by Hasan et al. about the higher awareness of women about HIV/AIDS parallels the findings of the present study (22). Contrary to this, the observations of Chory et al. and Nabunya et al. imply that women have poorer knowledge than men (23, 24). Periodontists demonstrated the highest level of comprehension among the different dental specialists, followed by oral medicine practitioners. Conversely, dental hygiene students and dental hygienists displayed the lowest level of comprehension. The investigation by Lima et al. further points to students’ overall lack of HIV-related knowledge (25). Nasir et al. have also astutely highlighted the deficiency in students’ knowledge, which renders them unprepared to effectively treat HIV patients (26). Additionally, a Saudi Arabian study by Alali et al. revealed unsatisfactory knowledge and a rather disappointing attitude by dental students toward matters pertaining to HIV (27). It comes as an enormous surprise that dental hygienists and dental hygiene students have such an alarming shortage of knowledge and comprehension about an assortment of facets of the periodontal and HIV relationship, including management and results. It is unanticipated from the professionals who hold crucial responsibilities in the delivery of periodontal care and are also deemed qualified to perform rapid HIV testing in dental settings, as per the report of Santella et al. (28).

The dental professionals, regardless of their specialty, exhibited very low knowledge and comprehension regarding the latest advancements concerning the significance of periodontal treatment on the course and outcomes of HIV infection. A longitudinal observation by Valentine et al., looked at how treating periodontal disease affected the HIV inflammatory profile, and revealed a notable enhancement in the CD4 counts of HIV patients when periodontal inflammation was successfully resolved (7). The prevailing belief among the dental professionals participating in the present study is that periodontal treatment has the potential to mitigate the progression of HIV infection, although no scientific data exists to substantiate this belief. It is noteworthy that the dental professionals had high comprehension of the impact of HIV on the exacerbation of preexisting periodontal diseases in individuals with HIV. Furthermore, the majority of them agreed that they learned about HIV-associated periodontal manifestations during their dental education. On the other hand, it was also acknowledged that their experience of providing periodontal care to individuals with HIV was limited and lacked theoretical knowledge in this specific domain. These deficiencies in comprehension and expertise may be a possible attribution for the continued occurrence of periodontal diseases in people with HIV, despite the use of HAART, as documented by Fricke et al. in their case–control research (29).

The research participants had very low comprehension regarding the safety of ultrasonic scaling in HIV patients. According to the Centers for Disease Control and Prevention (CDC), ultrasonic scaling in HIV patients is safe if standard precautions such as personal protective equipment (PPE), respiratory hygiene, sharp safety, and other infection control procedures are followed (30). It is surprising that the research participants have a low comprehension of the typical periodontal entities in HIV, such as linear gingival erythema, necrotizing gingivitis, and necrotizing periodontitis. Lugo et al. and Gomez et al. have identified linear marginal erythema as a prevalent progressive periodontal lesion linked to HIV, identified by an intense redness of the marginal gingiva (31, 32). Necrotizing periodontal disease is a periodontal condition frequently found in HIV patients (5). The insufficient comprehension among dental professionals may lead to diminished self-confidence, perhaps resulting in a reluctance to provide necessary periodontal care to HIV patients. Additionally, it could lead to insufficient and inappropriate periodontal care for these individuals since they may not identify the HIV-associated periodontal signs and treatment strategies for the same. Such a possibility has already been hinted at by the report authored by Olaniyi Olufemi Taiwo (33). In an investigation published by Dhanya et al., it is evident that dentists exhibit a certain degree of hesitancy to provide general dental treatment for patients infected with HIV when there is a deficiency in their understanding and proficiency in managing such cases (34).

The novelty of this topic in being the first to investigate comprehension about the periodontal-HIV link and a comparatively larger sample size that encompasses several dental professional specializations showcases the primary strengths of this research. The survey fatigue resulting from the extensive number of surveyed items could be a possible limitation of this questionnaire research. Another limitation is the small number of participants in the specialist subgroups, which limits comparisons between them.

The findings of the present study indicate that dental education is disproportionately emphasized on the general and manifestation aspects, with minimal attention given to the crucial HIV-linked periodontal management components, despite the fact that dental professionals primarily operate within a competency-based approach. The study outcome could potentially serve as an invaluable instrument for self-assessment by dental professionals and educators, as it reflects on the increasingly significant issue of periodontal and systemic diseases relationships. HIV/AIDS ought not to persist as an unspoken taboo or disregarded subject within the dental field, particularly in periodontics, but rather should be given attention and prominence in dental schools and professional development programs.

## Conclusion

Dental professionals are notably incomprehensive, unfamiliar, and lacking in expertise in the realm of periodontal facets of HIV. The periodontists and oral medicine practitioners showed a substantial amount of comprehension, while the dental hygiene students and dental hygienists presented a conspicuously inadequate level of comprehension.

## Data availability statement

The original contributions presented in the study are included in the article/supplementary material, further inquiries can be directed to the corresponding author.

## Ethics statement

This study was approved by the Qassim University’s institutional review board. Participants provided their consent to participate in the study.

## Author contributions

MA: Conceptualization, Data curation, Investigation, Methodology, Resources, Supervision, Validation, Writing – original draft, Writing – review & editing.
